# Human protein secretory pathway genes are expressed in a tissue-specific pattern to match processing demands of the secretome

**DOI:** 10.1038/s41540-017-0021-4

**Published:** 2017-08-18

**Authors:** Amir Feizi, Francesco Gatto, Mathias Uhlen, Jens Nielsen

**Affiliations:** 1 Department of Biology and Biological Engineering, Kemivägen 10, Chalmers University of Technology, SE41296 Gothenburg, Sweden; 2 Novo Nordisk Foundation Center for Biosustainability, Chalmers University of Technology, SE41296 Gothenburg, Sweden; 3 Novo Nordisk Foundation Center for Biosustainability, Technical University of Denmark, DK2970 Hørsholm, Denmark; 4 Science for Life Laboratory, Royal Institute of Technology, SE-17121 Stockholm, Sweden

**Keywords:** Systems analysis, Cell biology

## Abstract

Protein secretory pathway in eukaryal cells is responsible for delivering functional secretory proteins. The dysfunction of this pathway causes a range of important human diseases from congenital disorders to cancer. Despite the piled-up knowledge on the molecular biology and biochemistry level, the tissue-specific expression of the secretory pathway genes has not been analyzed on the transcriptome level. Based on the recent RNA-sequencing studies, the largest fraction of tissue-specific transcriptome encodes for the secretome (secretory proteins). Here, the question arises that if the expression levels of the secretory pathway genes have a tissue-specific tuning. In this study, we tackled this question by performing a meta-analysis of the recently published transcriptome data on human tissues. As a result, we detected 68 as called “extreme genes” which show an unusual expression pattern in specific gene families of the secretory pathway. We also inspected the potential functional link between detected extreme genes and the corresponding tissues enriched secretome. As a result, the detected extreme genes showed correlation with the enrichment of the nature and number of specific post-translational modifications in each tissue’s secretome. Our findings conciliate both the housekeeping and tissue-specific nature of the protein secretory pathway, which we attribute to a fine-tuned regulation of defined gene families to support the diversity of secreted proteins and their modifications.

## Gene expression: The human protein secretion pathway is tissue specific

The secretory pathway, an ubiquitous cellular machinery in human cells, is here shown to have tissue-specific characteristics. A research team led by Prof. Jens Nielsen at the Chalmers University of Technology performed a meta-analysis of the gene expressions of the secretory pathway’ component. They detected that even though most of these components are expressed in all tissues, there exist distinct components with fine-tuned expression. They further evaluated the functional link between the detected tuning and the processes that are demanded to make a set of tissue-specific proteins such as endocrine system hormones or their receptors. These findings open up a new avenue to understand the function of the secretion pathway in human tissues with possible applications for improving production of pharmaceutical proteins and getting more insight into the mechanisms underlying diseases such as diabetes that are connected with the endocrine system.

## Introduction

**Fig. 1:**
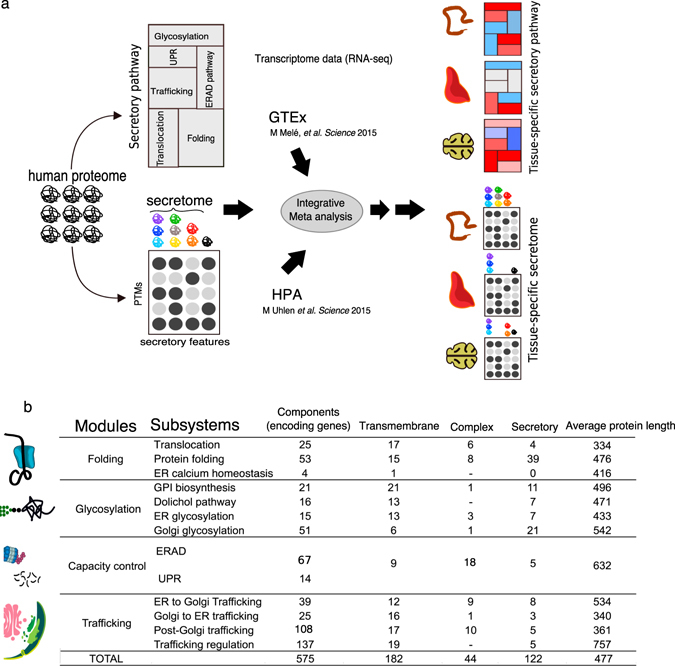
**a** The workflow of the analysis steps. As it has shown, we have reconstructed the secretory pathway network including different subsystem. In parallel, we have defined human secretome integrating the post-translational modifications (PTMs) information obtained from UniProt. Two independent transcriptome data including HPA and GTEx data sets were used for the meta-analysis to define the tissue-specific secretome and secretory pathways. **b** The properties of the general secretory pathway network. The secretory pathway network has 575 core components that are accommodated in four main functional modules and 13 subsystems. The complex column represent the number of the protein complexes in each subsystem. The secretory column shows the number of components that are the clients of the secretory pathway

In eukarya, the protein secretory pathway is an essential, efficient, and accurate molecular machinery for preparing and exporting proteins to expose the extracellular environment. This machinery includes various functional modules which are compartmentalized along the endoplasmic reticulum (ER) and Golgi apparatus. These modules are responsible for folding, processing of the post-translational modifications (PTMs), and trafficking of the proteins routed to the membrane of extracellular space.^1, 2^ In human, a functioning secretory pathway is essential for the body physiology. The majority of the hormones, peptidases, receptors/channels, extracellular matrix components, coagulation factors, transporters are all clients of this machinery.^2^ Unsurprisingly, dysfunction of the secretory pathway is the cause of a variety of systemic or developmental diseases, like cancer, diabetes, Parkinson’s disease, and congenital neurodegenerative disorders.^3–7^ The molecular biology and biochemistry of this pivotal pathway are well-studied for its core components.^8–10^ However, the knowledge how these components are expressed across tissues is lacking. Although, primary transcription is a key player in defining which genes has specific expression in certain tissue(s), yet, until recent advances in sequencing technologies it was not possible to measure the precise quantity of the RNA expression level in the genome scale.^11^ The recent studies based on RNA-sequencing (RNA-seq) have shown that human tissues exhibit unique transcriptional signatures that show stability even in postmortem sampls.^12^ The Genotype-Tissue Expression Project (GTEx),^13^ and the Human Protein Atlas (HPA)^5^ has been recently published as two independent and comprehensive RNA-seq data sources on 30 human tissues. In HPA study, one of the major conclusion of the paper was that the largest fraction of the tissue enriched transcriptome codes for the secretory proteins (secretome). The secretory pathway has evolved to process specific PTMs encoded in secretory protein. Among the PTMs, glycosylation, sulfation and adding GPI-anchored (glycosylphosphatidylinositol) are the major modifications. Each secretory protein has its composition regarding the PTMs type and number of the sites. Therefore, tissue-specific secretome implies in each tissue a different set of proteins with specific PTMs form enter to the secretory pathway. This further means, in each tissue, functional modules which are responsible for the processing of the PTMs types are faced with the particular load of the sites to processes which is different from other tissue. Borrowed from manufacturing world, if there is an input pressure on a particular operating module in a production pipeline, to release the pressure more processing units needs to be used in that specific modules. In the context of the secretory pathway, the response to the tissue-specific pressure on processing specific PTMs can be a fine tuning of the components expression in a particular functional module. In this study, we performed a meta-analysis approach utilizing the transcriptome data to detect such adjustment (Fig .1a). Also, we also examined whether the genes coding secretory pathway components indicate a change in their expression level in connection with the explained processing pressure. The results of this study advance the fundamental understanding of the tissue-specific function of the secretion pathway in human tissues. The findings can also possibly aid surpassing a long time standing challenges in biopharmaceutical protein production, since the current bottleneck in the production of human proteins is the functional difference between the host (e.g., CHO cells) and parent secretion system.^14, 15^

## Results

### Overall assessment of the expression level of secretory pathway genes in different tissues

Secretory pathway processes proteins in a stepwise manner. These steps include several functional modules such as translocation, folding and glycosylation. Each module involves a set of cooperative proteins, in most cases, encoded by the genes which belong to the same gene family. We previously have defined 169 components (proteins) of the secretory pathway in yeast, and we mapped them into the subsystems representing distinct functional modules^16^. Using a similar approach, we allocated our defined 575 core components of the human general secretory pathway into 13 subsystems (Fig. 1b, Table EV1). As earlier mentioned, the major focus of this study was to dissect the extent to which the expression levels of genes encoding the components of the secretory pathway are tuned over different tissues. Therefore, using available transcriptome data, we investigated the tissue-wise variations in the mRNA levels of these 575 genes. We used GTEx^13^ and HPA^5^ as the two independent and comprehensive RNA-seq datasets on 30 intersected human tissues. Both datasets have provided an unprecedented resolution on RNA levels in the tissues, and the correlation between their measurements has shown to be significant.^12^ We chose to analyze the GTEx data as the primary dataset because he it benefits from a careful experimental design with more tissues samples. The expression levels are normalized for different confounding parameters and variation sources such as individual, sex, and age. Although in the paper the authors have reported some variation depending on the individual, sex or age, however, we did not found for any of our analyzed gene lists from secretory pathway significant dependency on the individual, sex or age expression variation (Fig. S3). We used HPA data as a control.

Before analyzing the variations, we performed a descriptive analysis of the expression levels of the genes both in the secretory pathway and secretome based on the HPA gene expression categories. Therefore, we assigned the genes to the groups such as *expressed in all*, *tissue-elevated* and *tissue-enriched*.^5, 12^ Of all 575 secretory pathway components, ~75% (*n* = 435) belonged to the *expressed in all* category, while ~25% (*n* = 140) were in tissue-specific categories (such as *tissue-elevated* and *tissue-enriched*) (see the methods for the definitions, Table EV1). The distributions of the 435 genes expression (*expressed in all)* are similar in different tissues (in log_10_ FPKM) based on both GTEx and HPA data (median approximately equal to 10 FPKM (Fragments Per Kilobase Million)). The pancreas, skeletal muscle, heart, and liver slightly lower median expression (Fig. S1).

**Fig. 2:**
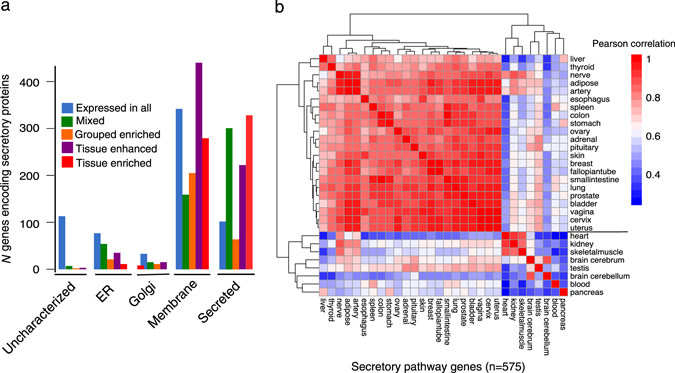
Expression of the secretory pathway and its clients across human tissues. **a** The frequency of the different expression categories of human secreted or membrane protein-encoding genes based on GTEx and localization information. Only protein with N-terminal signal peptide is included. Proteins were grouped according to localization. From this set, 503 proteins are localized in the lumen or the membrane of the ER and Golgi which encompass a large part of the secretory pathway components. **b** Shows the hierarchical clustering of cross-tissue correlation coefficients for the expression of secretory pathway genes in the “tissue-elevated” and “expressed in all” category (see the Methods for definition). The heatmap of the clustering for 30 human tissue pairwise correlation (Pearson correlations) is based on the expression profiles of 575 secretory pathway genes. The color code for correlation is depicted next to the heatmap

On the other hand, 10–20% of the transcriptome in human tissues (70% in the pancreas and salivary glands) translates into the secreted or the cell-membrane proteins. It has been shown that secretome holds the largest fraction of the tissue-specific proteome(Fig. 2a).^5^ In human proteome, 3328 proteins were predicted having an *N*-terminal signal peptide that dictates their entrance into the secretory pathway.^5^ From this group, 1218 are secreted proteins, and 1607 are cell membrane proteins.^5^ Contrary to the secretory pathway components, most of these proteins were assigned to the tissue-specific categories (e.g., *tissue enriched*) (Fig. 2a). This simple descriptive analysis indicates most of the secretory pathway genes are expressed in all tissues, while the secretome is tissue specific. Considering this, we contended that if the secretory pathway genes also follow any tissue-specific expression adjustment, despite its ubiquitous expression.

Analyzing the expression variations, as a preliminary assessment, we first checked for within-tissue variation of the secretory pathway genes as a whole machinery. Therefore, we performed a correlation analysis of the expression profiles of the genes encoding secretory pathway components across 30 tissues. As a result, interestingly, tissues were separated into the two groups. One group includes ~70% of all tissues (*n* = 23) showing medium to high correlation scores (*ρ* = 0.83 to 0.98). On the other hand, eight tissues including the pancreas, blood, kidney, skeletal muscle, heart, testis, and brain (cerebellum, and cerebrum) as the second group, showed low to medium correlation scores with the tissues in the first group (median coefficient *ρ* = 0.57 ± 0.17, permutation test *p* < 0.05). We repeated the analysis using HPA data and observed a similar clustering pattern (Fig. S2A). With a negligible effect of including the secretory pathway genes, the pancreas and the blood showed low cross-tissue correlation scores also at the whole transcriptome level. The weak correlation merits a potential confounding effect due to a deviance from their expression profile in these tissues (Fig. S2B). But, collectively, these results strengthen the idea of that opposite to the ubiquitous expression of the secretory pathway, at least in eight tissue there is a possible adjustment in the genes expression levels.

### Finding tissue-specific fine-tuning in secretory pathway gene families

Most of the subsystems in secretory pathway are comprised of several gene families. We showed earlier that eight tissues cluster away from other tissues because of the variations in the expression level of their secretory pathway’s genes (Fig. 2b). Therefore, to trace the differences causing these tissues to cluster away, we intended to reanalyze the correlations in the gene family level. Analyzing the variation in the gene family and subsystem level helped us to interpret the results in proper biological context. We identified 30 gene families with the size range between 4–44 gene members. These gene families are spread over different subsystems (summed up to 348 genes, see EV2 for detail). For example, post-Golgi trafficking (the largest subsystem) includes nine gene families including RAB family as the largest family (*n* = 72). RAB family genes encode for several different GTPase (diverged from the same ancestral origin^17, 18^) which are involved in vesicles trafficking from the ER to the Golgi and further down into the extracellular space. Reminding that most of the secretory pathway genes were assigned to the category *expressed in all* category (86%), the greatest fraction of all subsystems and corresponding gene families also assigned to this category (Fig. S4A, EV2). For instance, genes families in translocation subsystem all have their genes in *expressed in all* category. Interestingly, among 12 genes that have *tissue-enriched*, 11 genes are testis-specific proteins, and one gene (*CRYAA)* is a kidney-specific chaperone. The testis-specific genes spread to over ERAD (Endoplasmic-reticulum-associated protein degradation) (5 genes), protein folding (2 genes), Golgi glycosylation (2 genes) and trafficking regulation (2 genes) (Fig. S4B). For testis, as one the eight outlier tissues, these genes represent a specific expression for the secretory pathway. However, for the other seven tissues, there were no genes assigned to the *tissue-enriched* category Fig. S4B. We designed a 1110 pair-wise correlation analysis using the expression levels of each gene family expression profile across tissues. As a result, we observed each gene family showing specific correlations pattern across tissues.

**Fig. 3:**
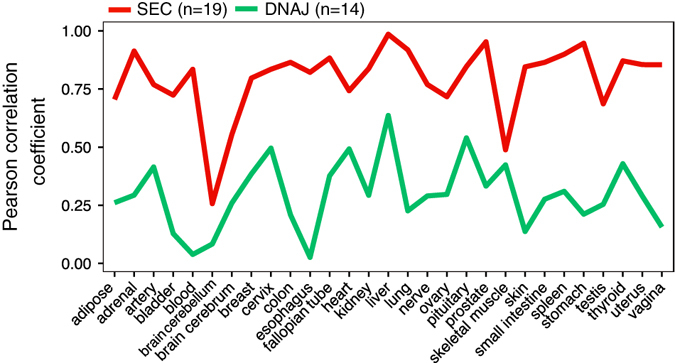
The correlations of the expression profiles from the selected gene families between the pancreas and other tissues. The Pearson correlation scores are shown as X-axis and tissues are located in *y*-axis. Each dot represents a correlation score of a specific gene family’s expression between the pancreas and another tissue. Each example gene family are depicted in different color. The color code for the gene families is located on the right side of the plot

To give an example, we take the expression levels of the five gene families in the pancreas, as one of the least correlated tissues (median *ρ* = 0.49 ± 0.11, Fig. 3). As you can see in Fig. 3, each of these gene families has their correlation pattern. For instance, the correlation scores for the SRP (signal receptor protein) and DNAJ (chaperone) gene families (involved in translocation and protein folding in ER) are low between the pancreas and most other tissues. On the other hand, the pair-wise correlations scores in most cases are high for the RAB or the SEC gene families (involved in membrane coat formation). It is remarkable to note that while the expression profile of secretory pathway in the pancreas as a whole machinery does do not correlate with most tissues (Fig. 2b), in gene family level, some gene families show a high correlation with most tissues. This evidently highlights the tuning of the expression profiles of the secretory pathway at gene family level rather than machinery as the whole. Among all correlation scores, ARF gene family (11 genes) shows high scores for most tissue-pairs. ARF (ADP-ribosylation factor) genes belong to the trafficking regulation subsystem and are involved in vesicle budding and uncoating within the Golgi apparatus^19^ (Fig. S5A). Conversely, the TBC (TBC1 domain family) gene family (19 genes) in more than 50% of the tissue pairs show low correlation (*R* < 0.6) (Fig. S5A and SB). TBC family genes are GTPase-activating proteins and are involved in the regulation of the vesicle trafficking.^20^ These results indicate in some of the particular gene families; the expression levels are modulated in a tissue-specific way.

### Identification of tissue-specific “extreme” genes

**Fig. 4:**
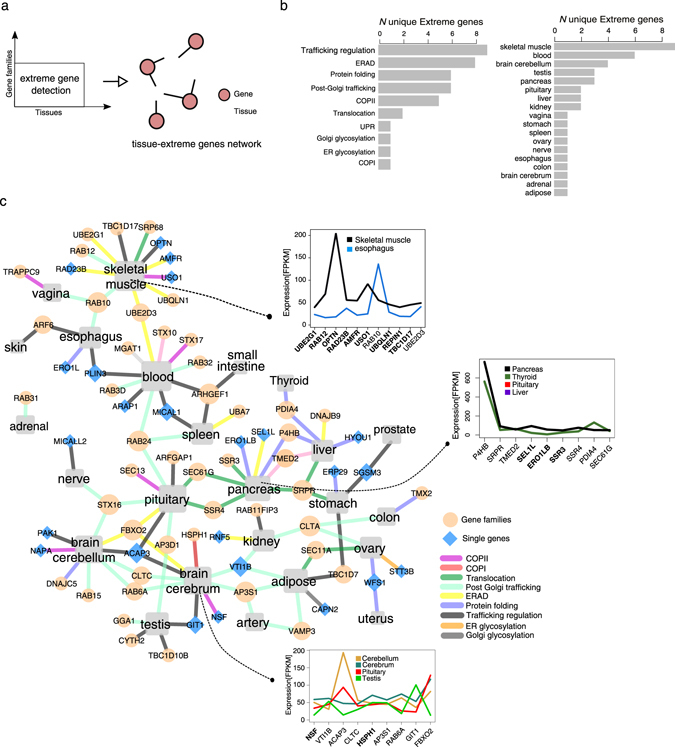
Detecting the tissue-specific extreme genes of secretory pathway genes families. **a** A simple workflow of the detection strategy of the extreme genes is shown. **b** shows the frequency of the extreme genes per subsystem and per tissue. **c** The represented network is the reconstructed tissue-extreme gene network. Extreme genes, whether as individual genes (*blue diamonds*) or as part of a gene family (*pale orange circles*), were grouped in the associated subsystem, and linked to each tissue (*gray box*) were they were detected as extreme. The detail expression level for three tissue (skeletal muscle, pancreas, and brain cerebrum) are plotted in the inboxes for their first neighbored detected extreme genes. All other colors not defined in the box refer to other tissue types

We showed that tissue-specific modulation in the expression levels of individual gene families could lead to the pair-wise low correlation scores for secretory pathway’s expression profile. In continue, we sought to identify the most extreme expression variations in gene families with low correlation scores. Therefore, for each gene family, we ran the Grubbs test^21^ to detect the outlier gene expressions, assuming that the total expression level of a gene family can vary among tissues (see Methods). We call the detected outlier genes as “extreme” genes, and we created a network of these identified genes connected to their corresponding particular tissue (Fig. 4a). This network visualizes which extreme genes from which gene families is specific or shared between any tissues (Fig. 4b). The detected extreme genes contribute the most to the low correlation scores calculated for each gene families across tissues (Fig. S5A). The tissues earlier were shown to cluster apart (Fig. 2b) have the largest set of detected extreme genes. These extreme genes are in the gene families which code the components for the subsystems like trafficking regulation, ERAD, protein folding and post-Golgi trafficking. Among the tissues, the Skeletal muscle has the largest number of the extreme genes (Fig. 4b). Noteworthy, for cross-validation, the genes with *tissue-enriched* category (from HPA) are also among the detected extreme genes. But, most of the extreme genes are marked as *expressed in all* category, therefore we instead suggest tissue-specific tuning for them.

As a separate validation, we compared our results with GTEx preferential expression analysis results. In the GTEx study, the authors performed a pair-wise differential gene expression analysis among tissue.^13^ The genes *q* > 0.99 (FDR = 0.01) and log2 fold change ≥4 in exclusive tissues were reported by them as tissue*-preferential genes*.^13^ Extracting preferential genes encoding secretory pathway components, in trustingly, we observed a large overlap with our detected extreme genes (Fig. S7). Meanwhile, we also checked for the *tissue-enriched* genes of the secretome to see if they are also reported as preferential genes. As a result, we found most of them are reported as preferential genes in GTEx study with top fold changes (Fig. S7). Some of these genes such as Leptin (LEP), insulin (INL), or prolactin (PRL) encode well-known secretory proteins, and their secretion has been studied for many years. This comparison made us confident on our method of detecting extreme genes that are assigned in *expressed in all* category.

Here we discuss some of the detected extreme genes in the three tissues with lowest correlation scores including the pancreas, skeletal muscle, and cerebrum (Fig. 4c). In the skeletal muscle and the pancreas, extreme genes that were uniquely associated with either of the two tissues, *OPTN* for skeletal muscle or *SEL1L* (involved in ERAD) for pancreas, showed an evident higher expression level (>10-fold change) comparing to the other tissues. It has been shown that *OPTN* plays a major role in the maintenance of the Golgi complex, in membrane trafficking and exocytosis, and it interacts with myosin VI and *Rab8*.^22, 23^ Surprisingly, *RAB12*, another extreme gene in the skeletal muscle, is shown to interact with *OPTN*.^24^ These findings suggest that even if secretory pathway genes were expressed rather ubiquitously in all tissues, specific tissues could spike the expression of specific genes in defined subsystems in a tissue-specific fashion. Reminding from our manufacturing example, now we could identify the units (extreme genes) that seem to be fine-tuned in a particular tissue. As next step, we, therefore, explored if these genes ultimate expression is correlated with enrichment of specific PTMs and functions in the secreted or membrane proteins specific in corresponding tissues.

### Tissue-specific enrichment in secreted and membrane proteins PTMs associated with expression tuning of the secretory pathway genes

**Fig. 5:**
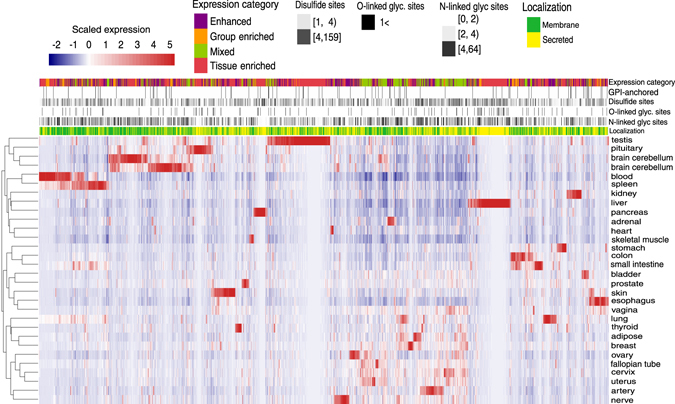
Hierarchical clustering of human tissue-specific secretome and membrane proteome expression and their PTMs. Each row represents a tissue and each column a tissue-specific secreted or membrane protein-encoding gene. *Z*-scores are scaled FPKM values for the expression of a given gene in a given tissue vs. other tissues. The annotation bars above the heatmap provide information regarding the expression category, number of disulfides, N-linked or O-linked glycosylation sites, GPI-anchored sites, and localization. The number of the sites are discretized to separate low, medium, and high number of PTM sites

To estimate the PTMs enrichment in each tissue, first, we had to define the tissue-specific secretome and membrane proteins. Therefore, we assembled a comprehensive list of 4098 genes encoding conventional (with signal peptide) (*n* = 3328) and unconventional (without signal peptide) (*n* = 680) secreted or membrane proteins (shown in Fig. 2a). Then, we extracted the GTEx expression profiles of these genes and performed hierarchical clustering of the tissues based on their expression correlation matrix (Pearson correlation). We limited this analyses to the genes in tissue-specific categories based HPA (2047 genes). The heat map of the clustering results reveals the tissue-specific expression patterns of the secreted and membrane proteins (Fig. 5). The tissues such as pancreas, testis, brain, skeletal muscle or kidney that were clustered in the separate clade with low correlation scores (shown in Fig. 2b) also show clear and specific expression in their secretome and membrane genes (Fig. 5). Instead, rest of the tissues that clustered together in Fig. 2b with high correlation scores (e.g. colon, ovary, breast, or bladder) share a sizeable number of highly expressed secreted and membrane proteins. Also, the number of secreted or membrane proteins unique to each tissue has a broad dynamic range, which reflects the complexity of the secretory requirements differs in each tissue.

Next, to integrate the PTMs information, we obtained information from UniProt on the number of sites for *N*-glycosylation (NG), the disulfide bond (DS), *O*-glycosylation (OG), GPI-anchored (GP) for all of the defined tissue-specific secreted and membrane proteins. Then, we integrated this information with the clustering result (Fig. 5). In general, most of the tissue-specific secreted and membrane proteins are enriched with N-linked glycosylation and disulfide sites. Specifically, pancreas and pituitary secretome displayed a lower enrichment in N-linked glycosylation sites and highly enrichment in disulfide sites (Fig. 5). O-linked and GPI-anchored sites are enriched rather in specific tissues. For example, the liver secretome is enriched in O-linked sites, whereas brain sub-regions are enriched with GPI-anchored membrane proteins (Fig. 5).

**Fig. 6:**
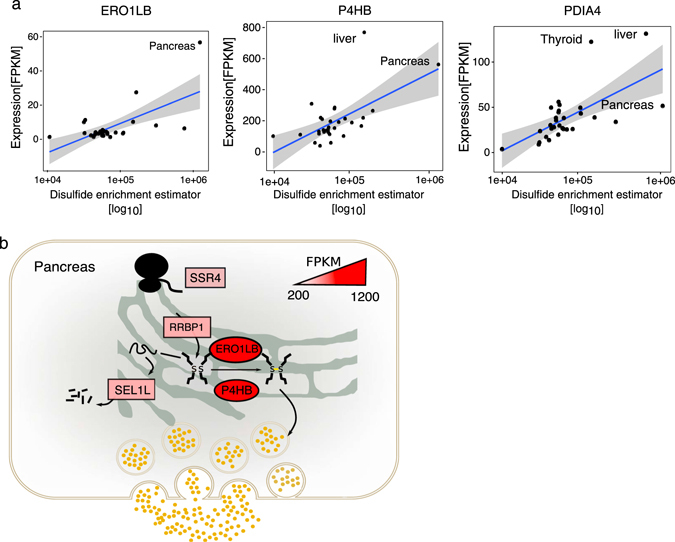
The link between the PDI gene family expression level and tissue-wise disulfide site enrichment. **a** The expression level (FPKM) of three detected extreme genes of PDI family (*ERO1LB, P4HB*, and *PDIA4*) are plotted against the calculated disulfide enrichment estimator. The linear regression lines are shown in blue with 95% of confidence interval. **b** *ERO1LB* and *P4HB* as detected extreme genes for PDI family in the pancreas are shown as eclipse, and their color is mapped to their FPKM values in the pancreas. The pancreas-specific extreme genes shown in rectangular boxes belongs to the gene families allocated to the translocation subsystem (*SSR4* and *RRBP1*) and ERAD (*SEL1L*)

Of the PTMs, we chose to explore the correlation between the disulfide sites load and the expression levels of the disulfide isomerase as processing components in each tissue. This is because of complexity of disulfide bond processing in less than other PTMs in secretory pathway regarding a number of the involved gene family and processing reactions. Therefore, to estimate the disulfide sites load on the secretory pathway in each tissue, we defined an enrichment estimator using the expression levels of proteins harboring disulfide sites as a proxy (see Methods). In brief, the estimator is a product function of the expression levels of secreted or membrane proteins and their corresponding number of the disulfide sites. We hypnotized higher estimator values to underscore a higher pressure on disulfide isomerases. Thus, higher values should correlate with the expression of the gene encoding the disulfide isomerase. Consistent with this hypothesis, we observed that the expression level of the PDI gene family, responsible for disulfide isomerase activity, linearly correlated with the disulfide enrichment estimator in each tissue (Fig. 6a). Strikingly, the expression level of *ERO1LB* gene, previously detected as a pancreas-specific extreme gene (Fig. 4c) was strongly correlated (*p*-value < 0.001) with calculated disulfide enrichment estimator in the pancreas (Fig. 6a). *ERO1LB*, an oxidoreductase involved in disulfide bond formation in the ER, is known to efficiently reoxidizes *P4HB*. *P4HB* is an enzyme which catalyzes the protein disulfide formation. Oxidation of *P4HB* by *ERO1LB* allow *P4HB* to sustain additional rounds of disulfide formation.^25^ We therefore also observed a correlation between the expression of *P4HB* in liver (a shared extreme gene by the liver and pancreas, Fig. 4c) and *PDIA4* (a shared extreme gene by the liver and thyroid, Fig. 4c) to the estimator values and found a positive correlation (*p*-value < 0.001 for *P4HB* and *p*-value < 0.01 for *PDIA4*) (Fig. 6a). These observations are clear evidence which suggests the tissue-specific fine-tuning of the PDI family expression level in response to the enrichment of the disulfide sites. As an experimental validation, we found a recent report in the literature that the expression level *ERO1LB* is precisely regulated in the pancreas.^26^

## Discussion

As we mentioned in the introduction, Uhlen et al. (2015)^5^ has recently shown that secretome is the largest fraction of the tissue-specific proteome. However, among the genes coding of the secretory pathway components which process and deliver the secretome proteins, only a small fraction (13%) found to be selectively expressed in certain tissues (mostly in testis). We reasoned that expression of secretome in tissue-specific way could put different PTMs processing pressure on secretory pathway subsystems which are responsible for processing the corresponding PTMs types. Searching for the footprints of this pressure, we detected expression spikes in individual members of gene family members in particular tissues. Detecting this kind of expression modulation in most gene-centric expression analysis such as differential expression analysis in difficult, while considering all members of a gene family expression gives us insight in the expression adjustment across various tissues. Gene expression is a dominant form of biological regulation that contributes to conferring tissue-specific functionality to diverse cell processes. It has previously shown by Kaessmann lab that purifying selection plays a key role in shaping the evolution of gene expression levels in mammalian organs^27^. Therefore, the tuning of the gene expression in each tissue evolutionary is independent of other tissues. The physiological and phenotypic demands have been the primary driving selection pressure on tissues. Secretory pathway has many gene families, and this indicates its function and complexity has been evolved through many gene duplications and neofunctionalization. So, it is not irrational for the cells to overexpress a specific member of a gene family to release the pressure caused by high processing load in a specific tissue. We could show this clearly in the case of the PDI gene family (Fig. 6a). Our result suggests that the expression levels in PDI gene family are tuned in respect to the processing load of disulfide sites (inside ER) in each tissue.

However, we do not assume this as the only source for the tissue-specific function of the secretory pathway, and signaling pathways, regulatory loops, and biological interactions are still important players.^28, 29^ On the other hand, drawing this conclusion is not trivial for other PTMs, because a large fraction of UniProt information on PTMs is based on the computational prediction; therefore, it includes a certain degree of false positives. Despite the recent advances, databases to serve tissue-specific information on N-linked or O-linked glycosylation are lacking. Also, it has been shown that even for a protein with experimentally detected glycosylation sites the glycoforms can be very heterogenic. Therefore, it is not easy to correlate the number of detected size with the processing load.^30–32^ The gene family size is another problem, for example despite PDI gene family, other genes families in secretory pathway are large, and therefore, it is more complex to link the detected extreme genes with specific processing load. For example, RAB gene family which is one of the human largest gene family has been studied comprehensively from the evolutionary and molecular point of view. However, due to their complex interaction network and complex function, more analysis and experiment design are needed to validate and understand why some members of this family have extreme expression level in an individual tissue. Although the experimental validation of our results remained to be explored, we found one external validation for *TMED2*, shared extremely gene between the liver and the pancreas (Fig. 4c). In a recent study, *TMED2* is shown to be a pancreas-specific protein,^33^ and it plays a critical role in cargo detection from ER (COPII vesicle) and the regulation of exocytic trafficking from the Golgi to the plasma membrane.^34–36^ All in all, these results shed light on important fundamental cross-tissue differences in the expression levels of the genes coding the secretory pathway’s component. A key question which remains to be explored is whether tissue-specific fine-tuning is the result of tissue specialization through evolution or the presence of regulatory programs specific to each tissue to fine-tune the control of its secretory pathway. This knowledge will empower us to boost our understanding of important diseases linked to the secretory pathway function in human and, on the other hand, to design better heterologous proteins expression host for biotechnological production.

## Methods

### Data collection

#### Transcriptome

We obtained the FPKM values for the human tissues from the analysis that has been performed by Uhlén et al. between^12^ on comparing the recently published RNA-Seq data generated by the Genotype-Tissue Expression (GTEx) consortium^13, 37^ and HPA consortium.^5^ In these datasets cutoff of 1 FPKM is used to indicate the presence or absence of transcripts for each gene in a tissue. We also used the categories defined in their paper. All human protein-coding genes were classified into one of six categories based on the FPKM levels in 32 tissues: (1) “Not detected”: FPKM < 1 in all tissues; (2) “Tissue enriched”—at least a 5-fold higher FPKM level in one tissue compared to all other tissues; (3) “Group enriched”—5-fold higher average FPKM value in a group of 2–7 tissues compared to all other tissues; (4) “Expressed in all tissues”—detected in all 32 tissues with FPKM >1; (5) “Tissue enhanced”—at least a 5-fold higher FPKM level in one tissue compared to the average value of all 32 tissues; (6) “Mixed”—the remaining genes detected in 1–31 tissues with FPKM >1 and in none of the above categories. We used the GTEx data sets as the main expression datasets in our analysis, which its measurements are for 20344 genes across 32 human tissues. The GTEx data is based on measurements for 1641 samples from 175 individuals representing 43 sites: 29 solid organ tissues, 11 brain sub-regions, whole blood, and two cell lines: Epstein–Barr virus–transformed lymphocytes (LCL) and cultured fibroblasts from the skin.^13^ The data from HPA^5^ were used in parallel to analyze the consistency. Interactome data: For protein–protein interaction data, we used the CCSB database for humans generated by Rolland et al. (2014),^38^ which includes ∼14000 high-quality binary protein–protein interactions. Protein complexes*:* Protein complex information retrieved from a census of human soluble protein complex data generated by Havugimana et al. (2012),^39^ which is a network of 13993 high-confidence physical interactions among 3006 stably associated soluble human proteins.

### Data processing, correlation analysis and visualization

We used recurrently “plyr,” “tidyr,” and “dplyr” R (https://www.r-project.org/) packages for all data processing steps and correlations analysis. The “pheatmap” and “ggplot2” packages used for visualization of the clustering results and plotting.

### Detection of the extreme genes

To detect the extreme genes in each gene family we used the Grubbs test^21^ using “outliers” package in R and GTEx as genes expression level source. The core formula of the calculated G-statistic for Grubbs test for each gene families is:

1 $$G = \frac{{\max \left| {{\it{{\rm X}}} - {\it{\bar {\rm X}}}} \right|}}{s}$$

Where with $\bar X$ and *s* denotes the sample mean and standard deviation, respectively. The Grubbs’ test statistic is the largest absolute deviation from the sample mean in units of the sample standard deviation.^40^

The outliers (extreme genes) are collected for all the gene families across tissues by filtering them based on an inbuilt two-sided test with calculated *p*-values <0.05. The Grubbs test assumes the input data has a normal distribution; however, the gene expression in the gene families violate this assumption. To avoid the false positives in the detection, we repeated the run by using HPA as independent expression resource. The output converted to a binary matrix of tissues-extreme genes and visualized as a network in Cytoscape.^41^

### Defining human secretory pathway

To collect the core components of the human secretory pathway, using the biomart package in R, first, we obtained the orthologs of 163 components of our previously reconstructed secretory pathway model in yeast.^16^ Also, the additional components were added up to 575, based on collecting relevant components from a comprehensive literature survey and KEGG secretion-related pathways including *protein processing in the endoplasmic reticulum* (ko04141) and *SNARE interactions in vesicular transport* (ko04130) (EV1). We defined 13 subsystems (Fig. 1a) based on the overlapping functions of the components adopting from our previously work on yeast secretory pathway genome-scale model.^16^ The Genes in each subsystem further classified into 30 gene families based on their nomenclature. The gene families consist of 347 genes and serve the core functional core of each subsystem. The rest of the secretory pathway genes are spread in different subsystem as functional units along with gene families (EV3, EV1).

### Defining the human secretome

We parsed the human UniProt GFF file and extracted the selected seven secretory features for the human proteome, including the following: *Signal Peptide, N-glycosylation sites, O-glycosylation sites, Disulfide bond, GPI-anchored, Transmembrane domain, Localization*. The obtained PTMs information was used to build a protein-specific information matrix. Each column of the matrix represents a specific PTMs type and each row belongs to a specific secretory protein. To define the tissue-specific enrichment of the different PTMs types, we integrated the constructed PTMs information matrix with the correlation analysis of the expression profiles from the genes encoding the secretome and membrane proteins. Among analyzed proteins as secretory proteins, 1242 proteins were without predicted signal peptide which 680 of them predicted to be secreted by unconventional secretion (secretome P NN-score >0.6). (Bendtsen et al. 2004 and Nickel & Seedorf, 2008) (EV2). We excluded these proteins from the analysis. For the clustering and visualization of the heatmaps corresponding to the secretome expression data (Fig. 6), we used the ComplexHeatmap packages.^42^

### Disulfide enrichment score

To be able to compare tissues for the enrichment of the disulfide sites in their secretory load we defined a disulfide enrichment estimator *DS*_*e*_ for each tissue to be as:

2 $$D{S_e} = log10\left({\mathop {\sum }\limits_{i = 1}^n fpk{m_i} \cdot \,\,d{s_i}} \right)$$

Where *i* is the number of the genes that are secreted or membrane proteins from 1to *n*, *fpkm*_*i*_ is the FPKM expression value of gene *i* and *ds*_*i*_ is the number of the disulfide sites in corresponding coded protein.

### Data availability

The HPA data used in the analysis is available from the original paper supplementary files (DOI: 10.1126/science.1260419) (ref. 5) and their download section in their database (http://www.proteinatlas.org/). The GTEx data used in the analysis is available from the original paper supplementary files (DOI: 10.1126/science.aaa0355) (ref. 13). The HPA and GTEx comparison data is available at the published papers supplementary files (DOI 10.15252/msb.20155865) (ref. 12). All the PTMs data for human proteome are available at UniProt data base GFF file for human proteins (http://www.uniprot.org/).^43^ All the codes for the data analysis and visualization are available upon request.

## Electronic supplementary material

Supplementary table and figure legends

Supplementary Figure 1

Supplementary Figure 2

Supplementary Figure 3

Supplementary Figure 4

Supplementary Figure 5

Supplementary Figure 6

Supplementary Figure 7

Supplementary Table 2

Supplementary Table 3

Supplementary Table 1

## Electronic supplementary material

**Supplementary Information** accompanies the paper on the *npj Systems Biology and Applications* website (doi:10.1038/s41540-017-0021-4).
