# Supplementary material for: Human protein secretory pathway genes are expressed in a tissue-specific pattern to match processing demands of the secretome
Source: NPJ Syst Biol Appl. 2017 Aug 18;3:22. doi: 10.1038/s41540-017-0021-4 (PMC5562915; doi:10.1038/s41540-017-0021-4)
Supplement: Supplementary file 1 — Supplementary table and figure legends [file 41540_2017_21_MOESM1_ESM.docx]

**Supplementary figures and Tables legends**

**Supplementary figure 1.**Secretory pathway expression profiles have been compared between GTEx and HPA data sets using boxplots. The expressions are log_10_ of the FPKM values.

**Supplementary figure 2.**(A) The heatmap shows the clustering results of the correlations scores across tissues which are calculated from their secretory pathway genes expression profiles extracted from HPA RNA-seq data. (B) Clustering of tissues based on their pair-wise correlation scores based on the whole transcriptome profile obtained from HPA RNA-seq data.

**Supplementary figure 3.**The contribution of tissue and individual to gene expression variation of PCGs (protein coding genes). The variation scores were calculated based on the original GTEx publication protocol (refer the text body for the reference to the publication). The color code for the data points indicates the genes which belong to the secretory pathway, secretome or other genes.

**Supplementary figure 4.**(A) The stacked barplot represents the number of the genes in each HPA defined expressed category (the color code in the right side) across different subsystem (*x-axis*) based on GTEx RNA-seq data. (B) The genes of the secretory pathway that are tissue enriched are summarized according to the detected tissue and the corresponding functional subsystem.

**Supplementary figure 5.**(A) The schematic representation for some of the secretory pathway’s gene family’s correlations scores histograms. The gene families are spread on their corresponding subsystem across the secretory pathway. The gene family name is depicted above each histogram. The color code in the bottom right shows the cut-off for high correlations and low correlations. (B) The histograms of the correlations scores for all secretory pathway’s gene families calculated based on pair-wise correlation analysis across 32 indicated (refer the methods part of the body text for more details).

**Supplementary figure 6.**(A) The dot plots show the log_2_ fold changes for the secretory pathway genes that are calculated based on GTEx original publication analysis on tissue-pairs differential expression analysis. The tissue names are shown in the x-axis, and the expression value is shown in the y-axis. The color the dots are mapped to the corresponding subsystems, and the shape of the dots are mapped to their expression category defined from GTEx data. The color codes are shown next to the plot. Some of the top expressed gene names are shown above the corresponding dots (B) The same data extracted instead for the secretome genes. However, the dot colors are mapped to the encoded genes protein localization data and the shape of the dots are mapped to their expression category. Some of the top expressed gene names are shown above the corresponding dots.

**Supplementary figure 7.**The scatter plots show the correlation between the disulfide enrichment estimator scores calculated for each tissue (refer to the methods section for more details) and the expression values (as FPKM) of the four genes involved in disulfide bridge formation in the ER protein folding subsystem.

**Supplementary Table 1.**

The human 575 core component of the Protein Secretory Pathway (PSP) with defined 13 subsystems (Subsystem columns), the HPA (32 tissues) and GTEx (30 tissue) expression values (fpkm) and categories.

**Supplementary Table 2.**human predicted secretome and membrane proteins including the HPA and GTEx expression and secretory features annotations (PTMs and localization).

**Supplementary Table 3.** The liver and pancrease tissue enriched genes including theit GTEx and HPA expression data and secretory features.
