# Supplementary figures and images for: Human protein secretory pathway genes are expressed in a tissue-specific pattern to match processing demands of the secretome
Source: NPJ Syst Biol Appl. 2017 Aug 18;3:22. doi: 10.1038/s41540-017-0021-4 (PMC5562915; doi:10.1038/s41540-017-0021-4)

FIGS1

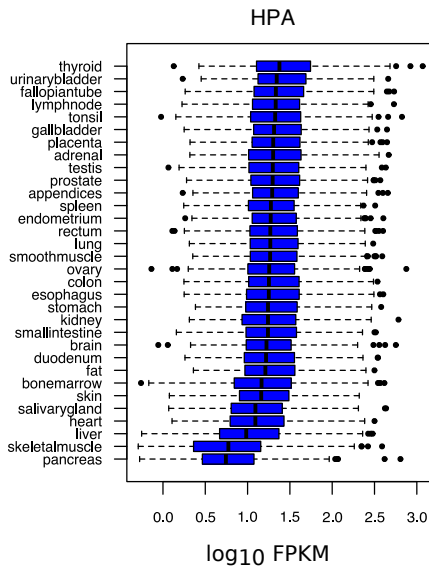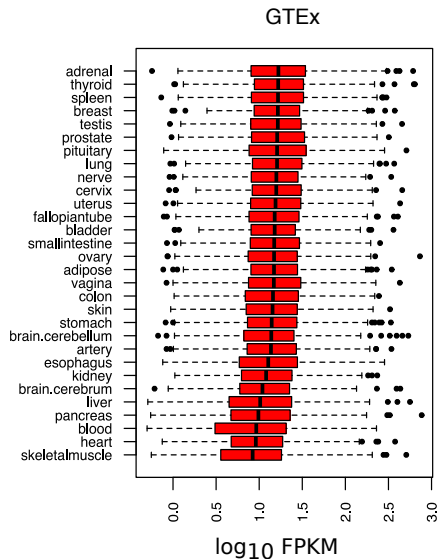

Supplement: Supplementary file 2 — Supplementary Figure 1 [file 41540_2017_21_MOESM2_ESM.pdf]

FIGS2

A

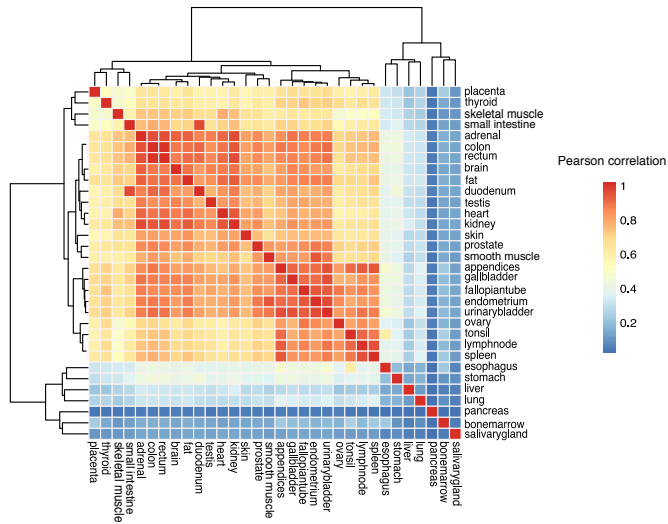

B

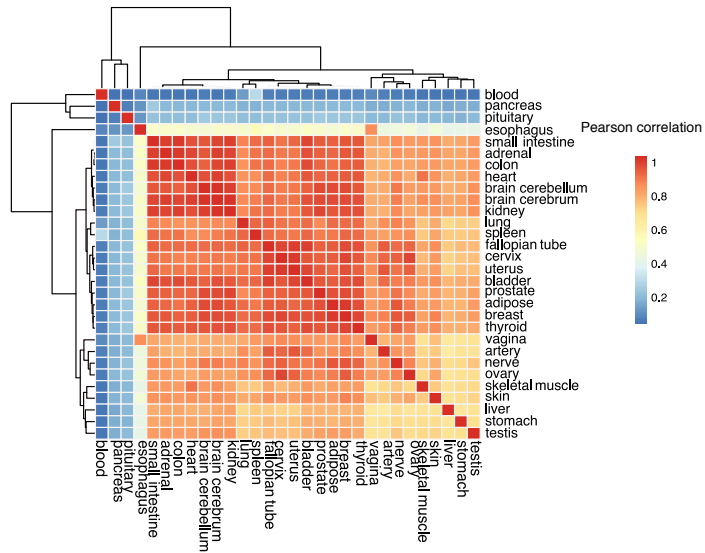

Supplement: Supplementary file 3 — Supplementary Figure 2 [file 41540_2017_21_MOESM3_ESM.pdf]

FIGS3

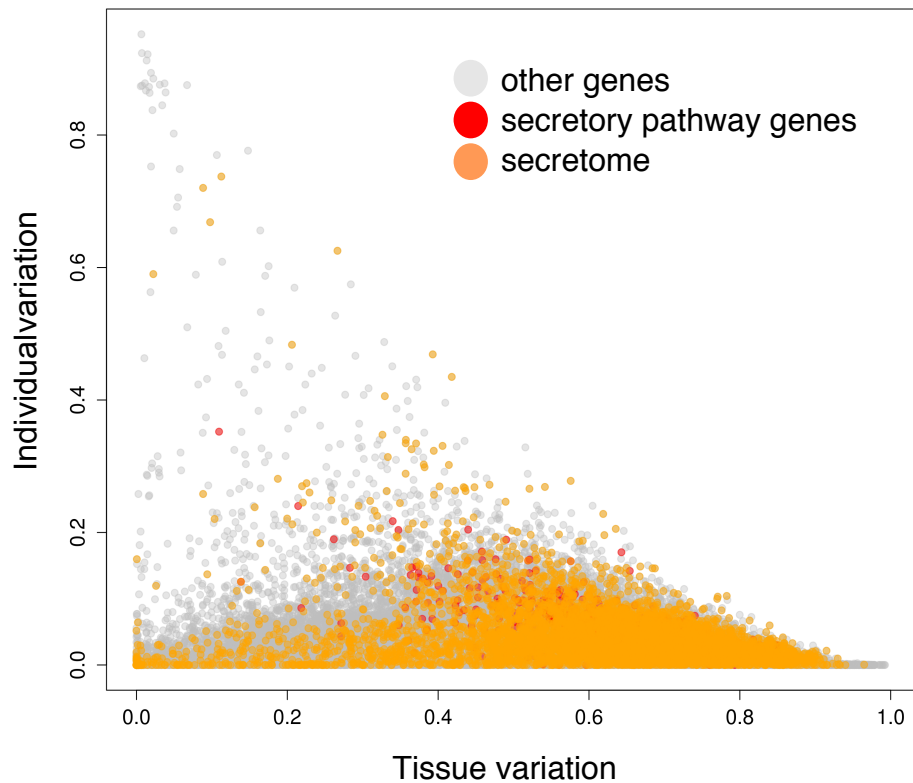

Supplement: Supplementary file 4 — Supplementary Figure 3 [file 41540_2017_21_MOESM4_ESM.pdf]

A

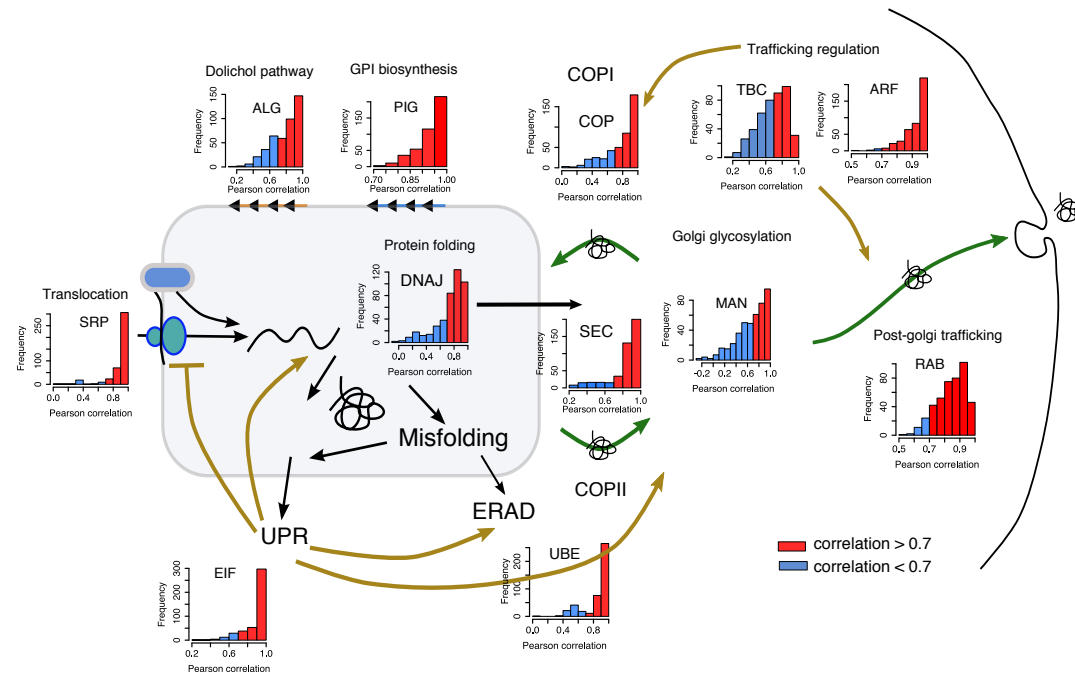

B

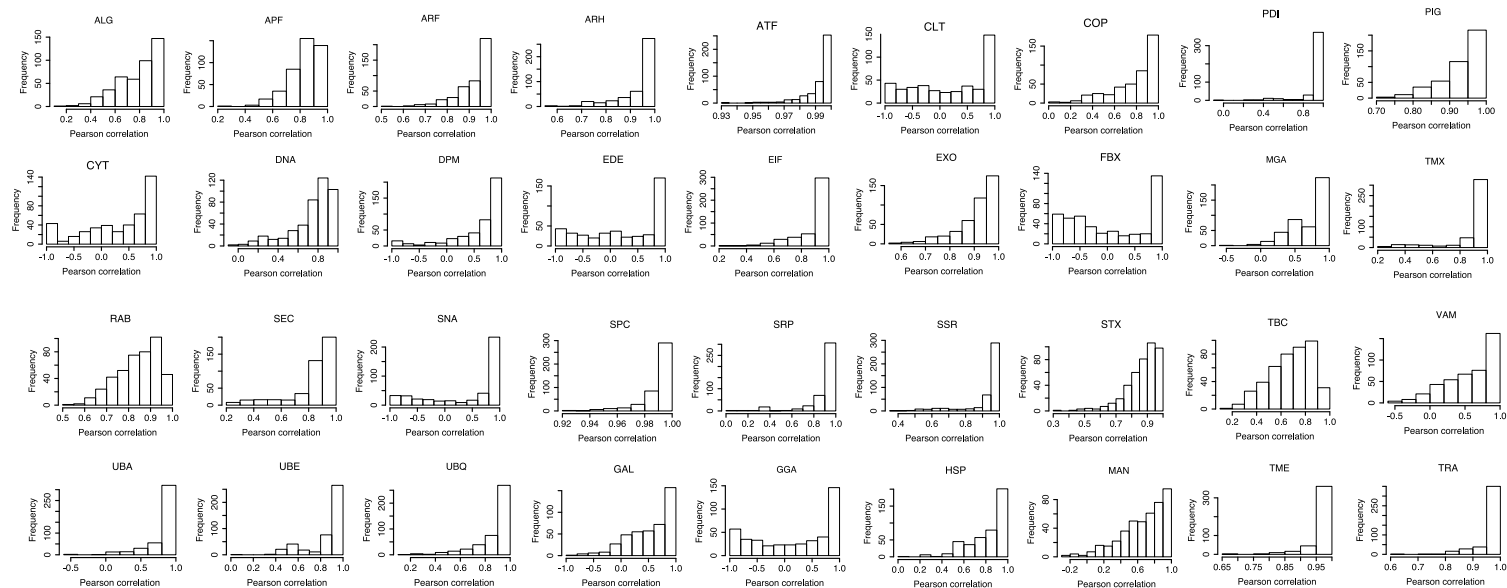

Supplement: Supplementary file 6 — Supplementary Figure 5 [file 41540_2017_21_MOESM6_ESM.pdf]

FIGS6

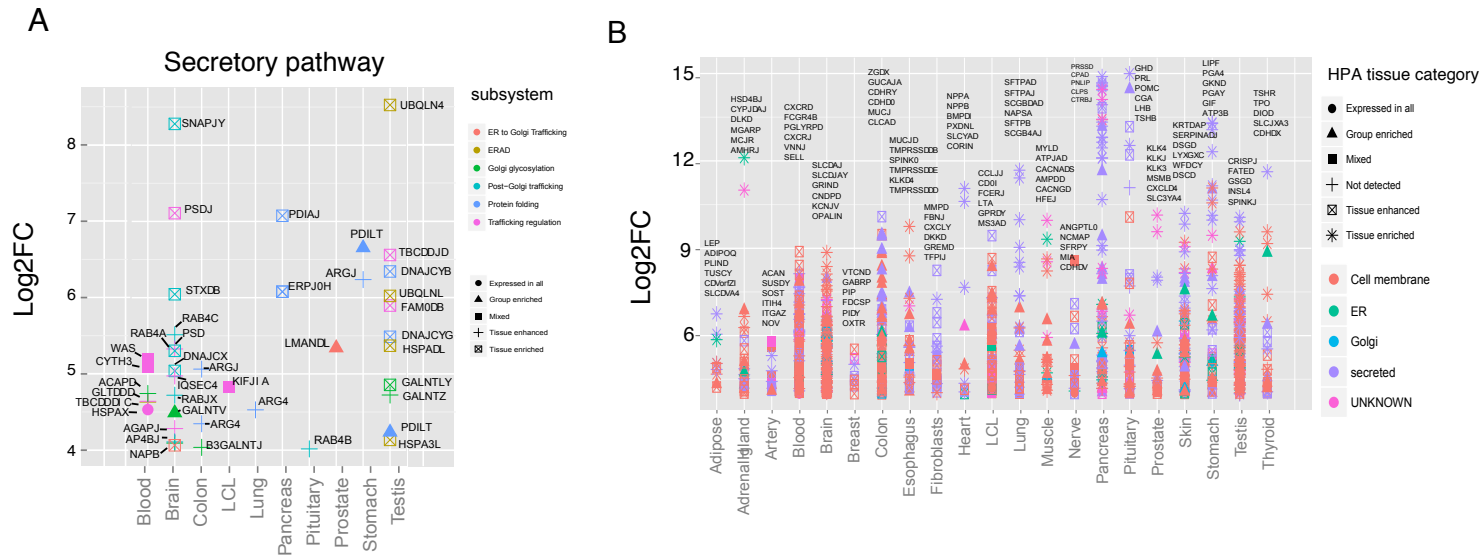

Supplement: Supplementary file 7 — Supplementary Figure 6 [file 41540_2017_21_MOESM7_ESM.pdf]

FIGS7

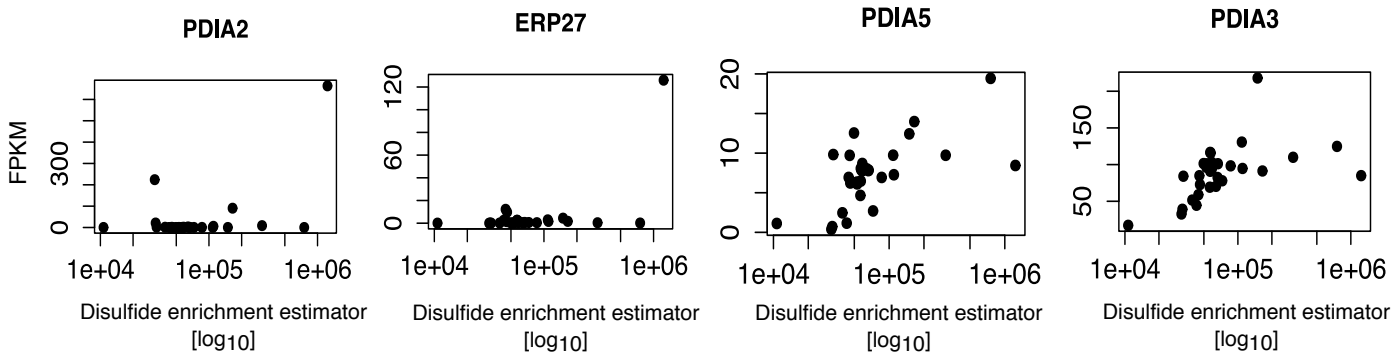

Supplement: Supplementary file 8 — Supplementary Figure 7 [file 41540_2017_21_MOESM8_ESM.pdf]
