# Supplementary material for: Human protein secretory pathway genes are expressed in a tissue-specific pattern to match processing demands of the secretome
Source: NPJ Syst Biol Appl. 2017 Aug 18;3:22. doi: 10.1038/s41540-017-0021-4 (PMC5562915; doi:10.1038/s41540-017-0021-4)
Supplement: Supplementary file 5 — Supplementary Figure 4 [file 41540_2017_21_MOESM5_ESM.pdf]

FIGS4

A

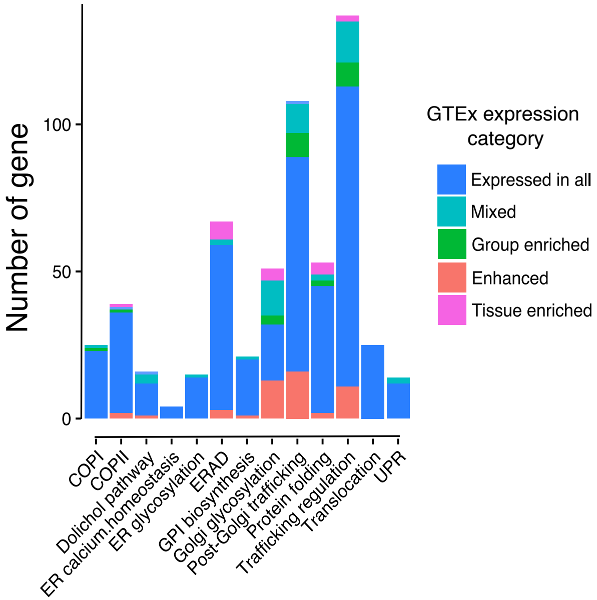

B

Gene	Tissue	Subsystem
CRYAA	kidney	Protein folding
UBQLNL	testis	ERAD
DNAJC5B	testis	Protein folding
TBC1D21	testis	Trafficking regulation
UBQLN3	testis	ERAD
ATXN3L	testis	ERAD
GLT6D1	testis	Golgi glycosylation
DNAJC5G	testis	Protein folding
HSPA4L	testis	ERAD
HSPA1L	testis	ERAD
GALNTL5	testis	Golgi glycosylation
FAM71B	testis	Trafficking regulation
